# TMF Attenuates Cognitive Impairment and Neuroinflammation by Inhibiting the MAPK/NF-κB Pathway in Alzheimer’s Disease: A Multi-Omics Analysis

**DOI:** 10.3390/md23020074

**Published:** 2025-02-07

**Authors:** Yonglin Liu, Xi Xu, Xiaoming Wu, Guodong Yang, Jiaxin Luo, Xinli Liang, Jie Chen, Yiguang Li

**Affiliations:** 1 National Key Laboratory for the Modernization of Classical and Famous Prescriptions of Chinese Medicine, Nanchang 330096, China; liuyonglin0595@163.com (Y.L.); paln7@163.com (X.L.); 2 School of Pharmacy, Jiangxi University of Chinese Medicine, Nanchang 330004, China; xixu200401@outlook.com (X.X.); wxm9849@163.com (X.W.); yang2428844581@163.com (G.Y.); ljx161802@163.com (J.L.); 3 Research and Development Department, Jiangzhong Pharmaceutical Co., Ltd., Nanchang 330103, China

**Keywords:** Alzheimer’s disease, 3′,4′,5,7-tetramethoxyflavone, neuroinflammation, transcriptomics, metabolomics, MAPK, NF-κB

## Abstract

The rising prevalence of Alzheimer’s disease (AD) underscores the urgent need for novel therapeutic agents derived from natural sources. Among flavonoids, 3′,4′,5,7-tetramethoxyflavone (TMF), a structural analog of luteolin, has gained attention for its favorable pharmacokinetics and potential neuroprotective properties. Despite the significant neuroprotective effects and favorable pharmacokinetics of TMF, its efficacy and mechanism of action in AD remain unclear. This study explored TMF’s pharmacological effects in AD models, highlighting its ability to improve memory and cognitive deficits in APP/PS1 mice. TMF reduced Aβ plaques, NFTs formation, and glial activation while suppressing neuroinflammation through the MAPK/NF-κB pathway. Further analysis in LPS-induced BV2 cells revealed TMF’s ability to reduce microglial activation. These findings highlight the anti-neuroinflammatory activity of TMF, suggesting its potential as a treatment for AD.

## 1. Introduction

Alzheimer’s disease (AD) is a progressive neurodegenerative condition marked by cognitive deterioration, memory loss, and personality changes [1,2]. AD poses a significant economic burden due to the high costs of symptomatic management and long-term care [3]. The pathogenesis of AD is complicated, and the scientific community believes it is related to Aβ hypotheses, tau protein hypotheses, and neuroinflammation [4]. Neuroinflammatory processes are pivotal in driving the progression of AD. Neuroinflammation is not merely a passive reaction to newly formed Aβ plaques and tau protein hypotheses; rather, it actively contributes to AD pathogenesis alongside Aβ plaques and tau protein hypotheses [5,6]. Therefore, modulating neuroinflammation offers a promising strategy for mitigating AD pathology.

Neuroinflammation acts as a double-edged sword in AD. While it aids in clearing Aβ deposits, it also generates cytotoxic substances that worsen AD pathology [7,8]. This inflammatory process is typically marked by reactive gliosis around amyloid plaques, with microglia as the primary inflammatory cells [9]. Microglia, which comprise approximately 10% of central nervous system (CNS) cells, become activated in AD by pro-inflammatory mediators, leading to neuroinflammation and neuronal damage [10,11]. Microglia activation further amplifies neuroinflammation by releasing pro-inflammatory cytokines, such as IL-1β, IL-6, and TNF-α. These cytokines stimulate the production of inducible nitric oxide synthase (iNOS), which accelerates Aβ deposition, resulting in excessive nitric oxide production and further neuronal injury [12,13]. Additionally, microglial activation triggers several key signaling pathways, including the MAPK and NF-κB pathways. NF-κB promotes microglial phagocytosis, cytokine secretion, and adaptive immune responses in neuroinflammation [14]. The MAPK pathway, involving P38 MAPK, JNK, and ERK, also plays a crucial role in microglial activation. The activation of MAPKs promotes amyloid precursor protein (APP) phosphorylation and increases Aβ accumulation [15,16]. These pathways can modulate microglial activation, suggesting that targeting the MAPK and NF-κB pathways could be promising strategies to control neuroinflammation and mitigate AD pathology [17]. Therefore, controlling neuroinflammation and inhibiting microglial activation are important strategies to ameliorate AD pathology.

Natural flavonoids hold considerable promise as therapeutic agents for managing neurodegenerative diseases, including AD. Luteolin, a naturally occurring compound found in the marine seagrass *Halophila stipulacea* and various land plants, has demonstrated significant potential in alleviating AD pathology [18,19,20]. It achieves this by reducing neuroinflammation, mitigating attention deficits, decreasing Aβ deposition, attenuating tau protein aggregation, improving mitochondrial function, and inhibiting hyperactivation of microglial and astrocyte cells. Previous studies indicate that luteolin’s derivative, 7,3′-disulfate, exhibits enhanced bioavailability and efficacy [21]. Similarly, the structural analog 3′,4′,5,7-tetramethoxyflavone (TMF) shows improved bioavailability, attributed to its methoxy groups [22,23,24]. In addition, TMF has demonstrated neuroprotective effects, and its gut-derived metabolites exhibit additional benefits in ameliorating AD pathology [25,26]. These findings underscore TMF’s potential to reduce neuroinflammation and offer therapeutic benefits in AD.

Although TMF has shown potential in alleviating neuroinflammation, its effects on AD pathology and associated neuroinflammatory processes remain inadequately understood. In this study, we utilized APP/PS1 mouse and BV2 cell models, integrating transcriptomics and metabolomics analysis to provide a comprehensive analysis of TMF’s impact.

## 2. Results

### 2.1. TMF Improved Memory Impairment in APP/PS1 Mice

**Figure 1:**
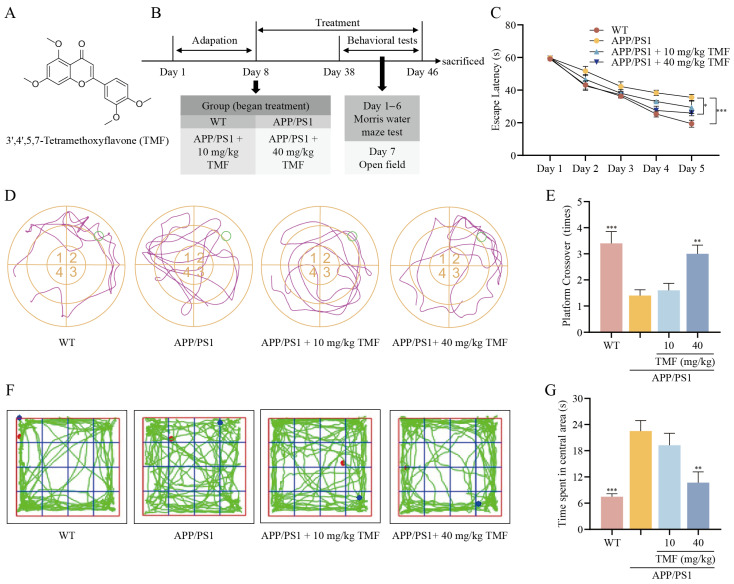
TMF ameliorates memory impairment in APP/PS1 mice. (**A**) Structural formulas of 3′,4′,5,7-tetramethoxyflavone (TMF). (**B**) Schedule of animal experiments. (**C**) Escape latency in the MWM probe trial. (**D**) Representative trajectories in MWM spatial exploration. (**E**) Number of platform crossovers in MWM spatial exploration. (**F**) Representing trajectories in OFT. (**G**) Time spent in the central area in OFT. Data shown as mean ± SEM, *n* = 10. * *p* < 0.05, ** *p* < 0.01 and *** *p* < 0.001 compared with APP/PS1 mice. One-way ANOVA and Tukey’s post hoc test were performed. And two-way ANOVA was performed to analyze WMW latency.

On day 5 of the MWM (Morris Water Maze) probe trial, nine-month-old APP/PS1 mice showed a longer escape latency compared to WT mice (*p* < 0.001), while the 40 mg/kg TMF group exhibited a marked reduction in escape latency (*p* < 0.05) (Figure 1C). During the spatial exploration period, the 40 mg/kg TMF treatment significantly reduced platform crossings by APP/PS1 mice (*p* < 0.01) (Figure 1D,E). In contrast, the 10 mg/kg TMF dose did not notably affect memory performance in MWM (*p* > 0.05). In an OFT (open-field test), APP/PS1 mice spent more time in the central area, indicating abnormal behavior, which was significantly reduced with 40 mg/kg TMF (*p* < 0.01). However, the 10 mg/kg dose had no significant impact on this behavior (*p* > 0.05) (Figure 1F–G). Overall, the 40 mg/kg TMF intervention was effective in improving memory impairment in APP/PS1 mice, while 10 mg/kg had no effect.

### 2.2. TMF Inhibited AD Pathology in APP/PS1 Mice

**Figure 2:**
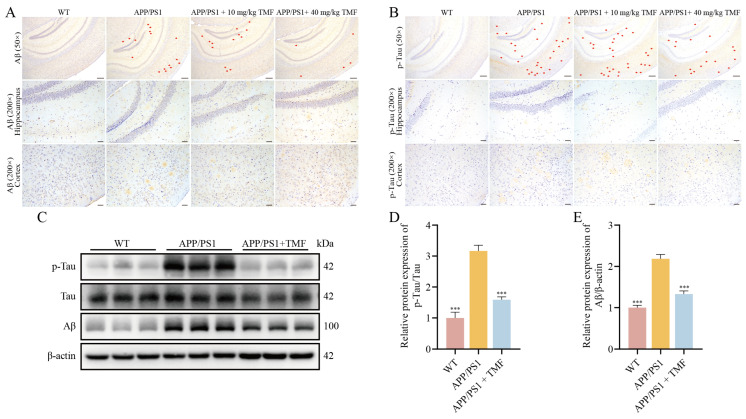
TMF ameliorates AD pathology in APP/PS1 mice. (**A**) The Aβ_1-42_ (red arrows) expression levels in mice brains. (**B**) The p-tau (red arrows) expression levels in mice brains. (**C**–**E**) Western blot analysis of Aβ and p-tau proteins in mice brains. Scale bars: 500 µm for 50× magnification and 100 µm for 200× magnification. Data are shown as mean ± SEM, *n* = 3. *** *p* < 0.001 compared to APP/PS1 mice. One-way ANOVA and Tukey’s post hoc test were performed.

To evaluate the effect of TMF on AD pathology in APP/PS1 mice, we used immunohistochemistry (IHC) to detect Aβ and p-tau levels in the mouse brain. Hippocampal and cortical Aβ and p-tau were reduced in 40 mg/kg TMF-treated mice compared to nine-month-old APP/PS1 mice. However, treatment with 10 mg/kg TMF reduced Aβ plaque accumulation in the cortex but increased plaque numbers in the hippocampus, with no significant changes in p-tau levels in the brain (Figure 2A,B). Similarly to the IHC results, Western blot analyses showed that Aβ and p-tau were inhibited by 40 mg/kg TMF treatment (*p* < 0.001) (Figure 2C–E). Combining the behavioral and AD pathology studies, we found that 40 mg/kg TMF had a significant ameliorative effect on AD pathology and, therefore, selected 40 mg/kg TMF for the follow-up study. These results suggest that TMF ameliorates AD pathology.

### 2.3. TMF Inhibited Neuroinflammation in APP/PS1 Mice

**Figure 3:**
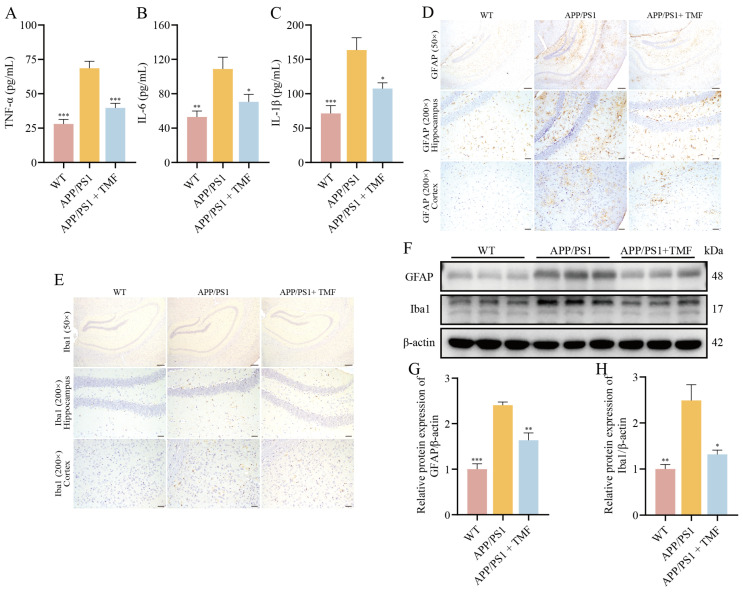
TMF ameliorates neuroinflammation in APP/PS1 mice. (**A**–**C**) Inflammatory factors TNF-α, IL-1β, and IL-6 in the mice brains. (**D**) The GFAP expression levels in mice brains. (**E**) The Iba1 expression levels in mice brains. (**F**–**H**) Western blot analysis of GFAP and Iba1 proteins in mice brains. Scale bars: 500 µm for 50× magnification and 100 µm for 200× magnification. Data shown as mean ± SEM, *n* = 3. * *p* < 0.05, ** *p* < 0.01 and *** *p* < 0.001 compared with APP/PS1 mice. One-way ANOVA and Tukey’s post hoc test were performed.

In nine-month-old APP/PS1 mice, levels of these cytokines were elevated, but TMF treatment reduced their expression (*p* < 0.05) (Figure 3A–C). Our findings indicate that TMF intervention lowered Iba1 and GFAP levels in APP/PS1 mice (*p* < 0.05), inhibiting microglia and astrocyte activation (Figure 3D–H). These results suggest that TMF suppresses neuroinflammation by inhibiting microglia and astrocyte activation.

### 2.4. TMF-Regulated Hippocampal Gene Expression in APP/PS1 Mice

**Figure 4:**
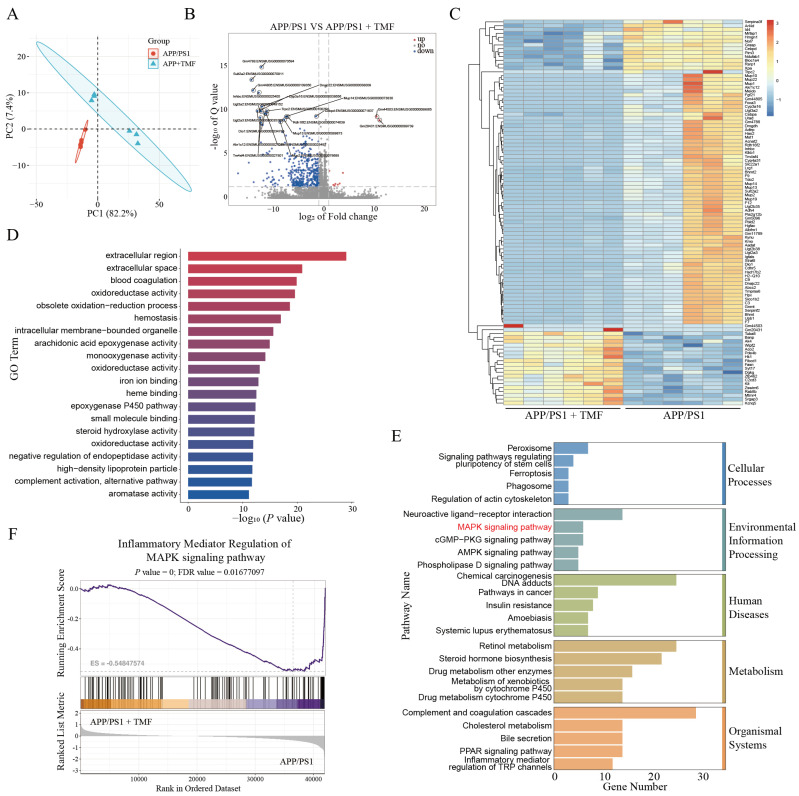
TMF alters mRNA expression in the hippocampus of APP/PS1 mice. (**A**) PCA analysis of mRNA expression. (**B**) Volcano plots of DEGs. (**C**) DEGs heatmap. (**D**) GO analysis of DEGs. (**E**) KEGG analysis of DEGs. (**F**) GSEA analysis of DEGs. *n* = 6. Student’s *t*-test was performed using DEGs.

To investigate how TMF ameliorates AD pathology, RNA-seq analysis was performed on the hippocampi of nine-month-old APP/PS1 and TMF treatment mice. Figure 4A shows that PCA analysis distinguished gene expression profiles between the two groups. Volcano plots highlighted 422 significantly differentially expressed genes (DEGs) (413 downregulated and 9 upregulated) between the two groups (Figure 4B). Heat map analysis further confirmed the overall downregulation of gene expression following TMF treatment (Figure 4C). Notably, genes associated with neuroinflammation, such as Cebpd, Cebpa, AK4, Kit, and Lrg1, showed significant changes in expression following TMF treatment [27,28,29,30,31]. The key TMF-inhibited pathways were investigated by GO and KEGG enrichment analysis. GO analysis reveals that these DEGs are enriched in a variety of functional categories (Figure 4D), and KEGG analysis identified enrichment in neuroactive ligand-receptor interaction, MAPK pathways, cGMP-PKG pathways, and AMPK pathways (Figure 4E). Further GSEA analysis showed that MAPK pathway expression was elevated in the APP/PS1 mice group but reduced in the TMF mice group (Figure 4F), suggesting TMF’s potential role in modulating the MAPK pathway.

### 2.5. TMF-Regulated Plasma Metabolic Profiles in APP/PS1 Mice

**Figure 5:**
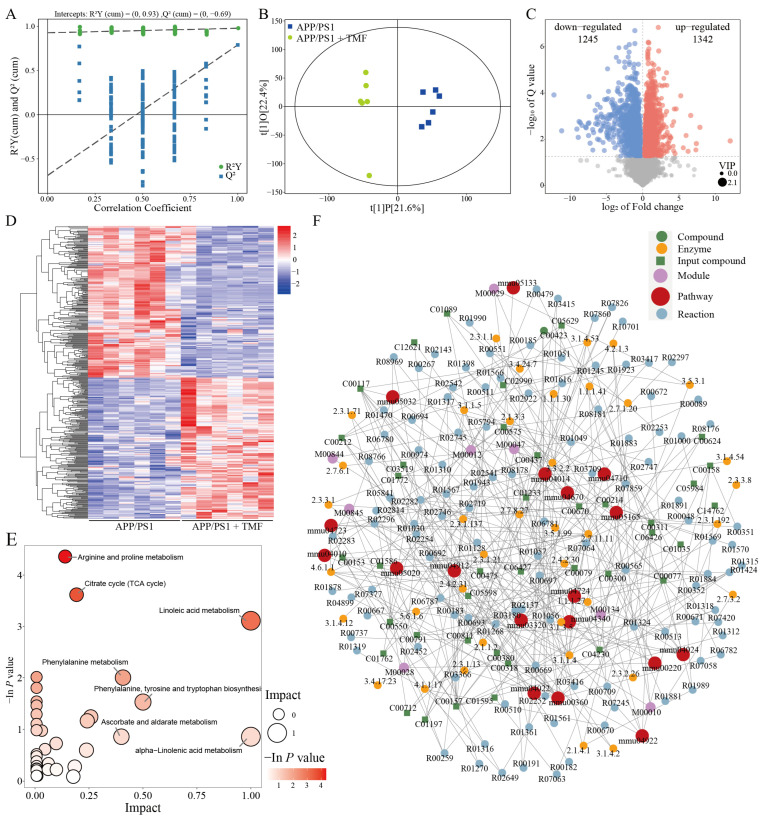
TMF alters the plasma metabolites of APP/PS1. (**A**,**B**) OPLS-DA analysis of plasma metabolites. (**C**) Volcano plots of DMs. (**D**) DMs heatmap. (**E**) KEGG analysis of DMs. (**F**) Network analysis of the interactions between pathways, modules, enzymes, reactions, and metabolites. *n* = 6. Student’s *t*-test was performed using DMs.

Predictive transcriptomics analysis suggested that TMF may influence the metabolism of APP/PS1 mice. Metabolomics was performed to assess the effect of TMF on their metabolic profiles. Metabolites between the two groups were assessed by OPLS-DA, and different metabolites (DMs) were identified with VIP > 1.0 and *p* < 0.05. As shown in Figure 5A,B, significant differences in plasma metabolites were observed between the nine-month-old APP/PS1 and TMF treatment mice, indicating that TMF altered these profiles. A total of 2587 DMs were identified, with 1245 downregulated and 1342 upregulated after TMF treatment (Figure 5C,D). KEGG analysis showed that DMs were primarily enriched in 34 pathways (Figure 5E), such as arginine and proline metabolism. Based on these findings, an interaction network was constructed linking pathways, modules, enzymes, reactions, and metabolites (Figure 5F), showing that TMF-regulated metabolites impacted 18 pathways, including arginine biosynthesis, phenylalanine metabolism, the PPAR signaling pathway, and the MAPK signaling pathway (the top 10 pathways are shown in Supplementary Materials Table S3).

### 2.6. TMF Inhibited Activation of Neuroinflammatory Pathways in APP/PS1 Mice

**Figure 6:**
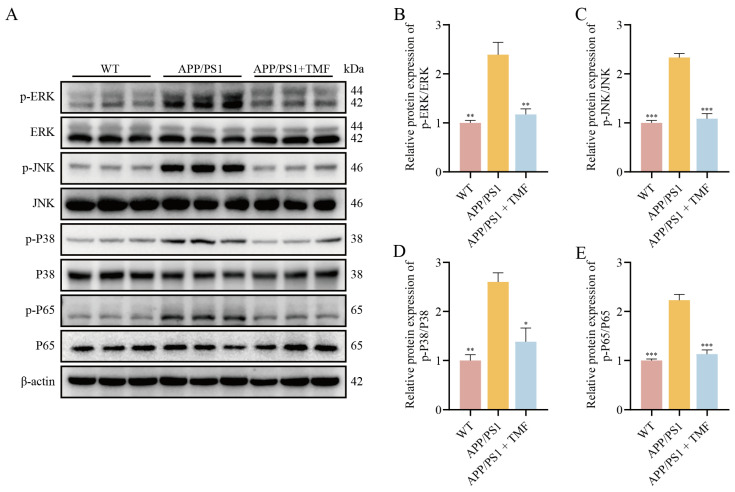
TMF inhibits the expression of the MAPK/NF-κB pathway in the brain of APP/PS1 mice. (**A**–**E**) Western blot analysis of the phosphorylation of ERK, JNK, P38, and P65 in mice brains. Data shown as mean ± SEM, *n* = 3. * *p* < 0.05, ** *p* < 0.01 and *** *p* < 0.001 compared with APP/PS1 mice. One-way ANOVA and Tukey’s post hoc test were performed.

Transcriptomics analysis identified the MAPK pathway as a key mechanism by which TMF may improve AD pathology, while metabolomics results underscored its role in managing metabolic dysregulation. Based on multi-omics results, we speculated that TMF could inhibit the activation of the MAPK pathway. To investigate this, we assessed the expression of key proteins ERK, JNK, P38, and the downstream NF-κB P65 protein by Western blotting. The results showed that the phosphorylation levels of ERK, JNK, P38 MAPK, and NF-κB P65 were higher in the nine-month-old APP/PS1 mice (*p* < 0.01), and TMF treatment reduced these levels (*p* < 0.05) (Figure 6). These results suggest that TMF suppresses neuroinflammation by inhibiting the activation of the NF-KB/MAPK pathway.

### 2.7. TMF Inhibited LPS-Induced Neuroinflammation in BV2 Cells

**Figure 7:**
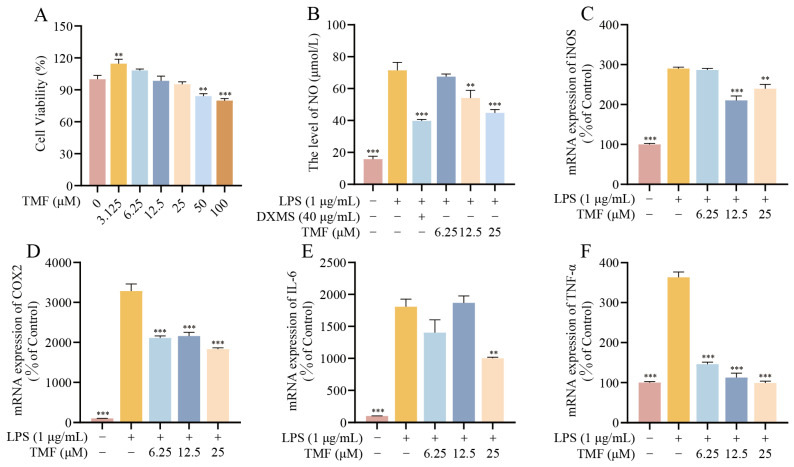
TMF alleviates the expression of inflammatory factors in LPS-induced BV2 cells. (**A**) BV2 cells viability. (**B**) The levels of NO. (**C**–**F**) mRNA expression levels of iNOS, COX-2, TNF-α, and IL-6. Data shown as mean ± SEM, *n* = 3. ** *p* < 0.01 and *** *p* < 0.001 compared with the LPS-induced BV2. One-way ANOVA and Tukey’s post hoc test were performed.

Animal studies demonstrated that TMF reduced neuroinflammation in APP/PS1 mice. The CCK-8 (Cell Counting Kit-8) assay assessed the cytotoxicity of TMF and showed that concentrations of 6.25 to 25 μM did not affect the viability of BV2 cells (*p* > 0.05), whereas 50 and 100 μM were cytotoxic (*p* < 0.01) (Figure 7A). Based on these findings, the non-toxic concentration range of 6.25 to 25 μM was selected for further research. To evaluate the impact of TMF on microglial activation and neuroinflammation, the inflammatory factor NO was quantified, and the mRNA expression levels of iNOS, COX-2, TNF-α, and IL-6 were analyzed using RT-qPCR. The results indicated that LPS (lipopolysaccharide) stimulation elevated NO levels in BV2 cells (*p* < 0.001), while TMF reduced NO concentrations (*p* < 0.01) (Figure 7B). Similarly, LPS exposure markedly increased the mRNA expression of inflammatory markers iNOS, COX-2, TNF-α, and IL-6, but these effects were mitigated by TMF intervention (*p* < 0.01) (Figure 7C–F). These findings suggest that TMF effectively inhibits microglia-mediated neuroinflammation.

### 2.8. TMF Inhibited Activation of Neuroinflammatory Pathways in LPS-Induced BV2 Cells

**Figure 8:**
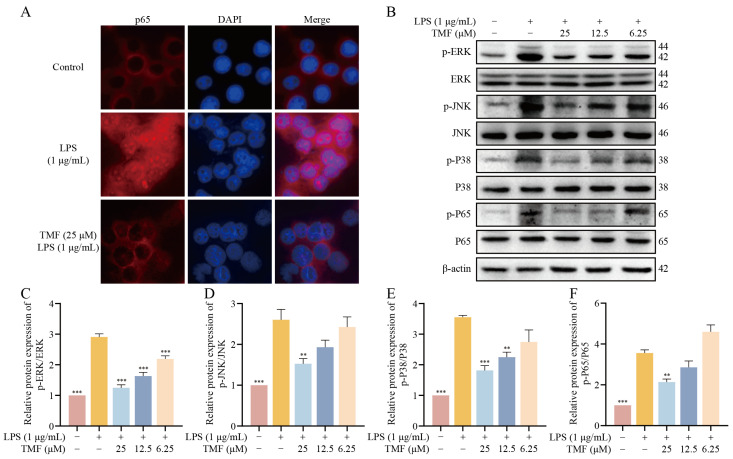
TMF inhibits the expression of the MAPK/NF-κB pathway in LPS-induced BV2 cells. (**A**) IF analysis of the levels of NF-κB P65. (**B**–**F**) Western blot analysis of the phosphorylation of ERK, JNK, P38, and P65 in BV2 cells. Data shown as mean ± SEM, *n* = 3. ** *p* < 0.01 and *** *p* < 0.001 compared with the LPS-induced BV2. One-way ANOVA and Tukey’s post hoc test were performed.

As previously described, we hypothesized that TMF inhibits the activation state of microglia by modulating the activation of the MAPK/NF-κB pathway. Immunofluorescence (IF) results showed that NF-kB p65 accumulated in the nuclear BV2 cells after LPS induction, and the nuclear accumulation of NF-kB p65 decreased after TMF treatment (Figure 8A). As shown in Figure 8B–F, LPS stimulation significantly upregulated the phosphorylation levels of ERK, JNK, P38, and P65, whereas TMF intervention significantly reduced their phosphorylation levels (*p* < 0.01). Our results suggest that TMF inhibits microglia-mediated neuroinflammation through the MAPK/NF-κB pathway.

## 3. Discussion

AD is a multifaceted neurodegenerative condition primarily and is the leading cause of dementia worldwide [32,33]. Despite its prevalence, there are currently no therapeutic agents that can safely and effectively halt the progression of AD [32]. Existing treatments provide only symptomatic relief and are often accompanied by significant limitations. For instance, NMDA receptor antagonists can temporarily alleviate cognitive symptoms in early disease stages, but fail to halt or reverse progression and may cause neurological complications [34]. Similarly, cholinesterase inhibition inhibitors improve neurotransmitter function initially, but lose effectiveness over time and are associated with severe side effects, such as hepatotoxicity [35]. NSAIDs have been considered potential prophylactic agents due to their ability to reduce inflammation; however, their severe side effects preclude long-term use [36]. Therefore, there is an urgent need for the development of novel therapeutic agents for the treatment of AD.

Recent advances in pharmacological research have identified flavonoids as promising candidates for the treatment of AD [37]. Among these, TMF, a flavonoid derived from various natural products, stands out due to its favorable pharmacokinetics and significant bioactivity. TMF has been demonstrated to inhibit microglial activation and exert neuroprotective effects [26], but its specific role in AD pathology remained unexplored before this study. Using the APP/PS1 mice, this study demonstrated that TMF significantly reduced Aβ deposition and NFT formation and alleviated neuroinflammation and metabolic disorders. These effects were achieved by inhibiting the activation of the MAPK/NF-κB signaling pathway. Furthermore, in vitro experiments revealed that TMF effectively suppressed microglial activation and reduced neuroinflammation by targeting the MAPK/NF-κB pathway. These findings provide the first evidence that TMF modulates neuroinflammation and mitigates AD pathology.

The pathological hallmarks of AD include the abnormal accumulation of Aβ and NFT formation, both of which disrupt neuronal function and drive cognitive decline [38]. Reducing Aβ and p-tau can improve memory impairment [39]. In this experiment, we used APP/PS1 double-transgenic mice exhibiting neuropathological features of AD [40]. In APP/PS1 mice, which develop AD pathology and cognitive impairments, TMF treatment significantly improved memory and reduced anxiety-related behaviors. These findings indicate that TMF attenuates cognitive deficits and AD pathology.

Neuroinflammation is now widely acknowledged as a key contributor to AD pathology, primarily driven by the sustained activation of microglia in response to Aβ plaques and NFTs [41]. Activated microglia release pro-inflammatory cytokines that amplify neuroinflammation, promote tau hyperphosphorylation, and exacerbate Aβ deposition [41,42]. This inflammatory cascade is further amplified by the activation of astrocytes, which contribute to neurotoxicity and neuronal damage [43]. Naringenin has been shown to attenuate neuroinflammation in APP/PS1 mice by downregulating TNF-α and IL-1β [44] in our study. Similarly, TMF significantly reduced the expression levels of TNF-α, IL-1β, and IL-6 levels. To further investigate the role of TMF in mitigating neuroinflammation, we evaluated the hyperactivation of microglia and astrocytes by measuring their respective markers: Iba1 for microglia and GFAP for astrocytes [45]. TMF treatment significantly reduced the expression of these markers, confirming its ability to inhibit the over-activation of both microglia and astrocytes. These findings indicate that TMF alleviates neuroinflammation by suppressing microglial and astrocytic overactivation in APP/PS1 mice.

To further elucidate the mechanisms by which TMF alleviates neuroinflammation in AD, we utilized a multi-omics approach that combined transcriptomics and metabolomics. Transcriptomics facilitated the rapid identification of mRNA changes, enabling the screening of therapeutic targets [46]. Concurrently, metabolomics analyzed small molecule metabolites in biological samples, revealing the drug’s effects on metabolic processes and supporting mechanistic insights [47]. Transcriptomics analysis revealed that TMF significantly influenced gene expression in APP/PS1 mice, particularly by downregulating genes involved in the MAPK signaling pathway. Similarly, metabolomics analysis indicated that TMF disrupted the metabolism of arginine and proline—critical pathways for cellular energy production, redox balance, and signaling. Disruptions in arginine and proline metabolism lead to an imbalance in NO levels, intensifying neuroinflammation and oxidative stress [48,49], which in turn activate microglia and increase the production of pro-inflammatory cytokines, primarily via the MAPK and NF-κB pathways [14,42]. Pathway–metabolite interaction network analysis further highlighted the MAPK signaling pathway as an important mechanism by which TMF alleviates metabolic disorders. Our results showed that TMF intervention significantly reduced the phosphorylation of ERK, JNK, P38, and NF-κB P65. These findings underscore that TMF regulates neuroinflammation and metabolic disorders through the MAPK/NF-κB pathway.

To validate the effects of TMF on microglial activation, we established an LPS-induced BV2 microglia model in vitro, which replicates the neuroinflammatory processes observed in AD [50]. Our findings demonstrated that TMF markedly reduced NO production in BV2 microglia and suppressed the mRNA expression of key inflammatory mediators, including iNOS, COX-2, TNF-α, and IL-6. Additionally, Western blot analysis confirmed that TMF inhibited LPS-induced phosphorylation of proteins associated with the MAPK/NF-κB pathway, specifically P38, ERK, JNK, and NF-κB P65. These results indicate that TMF effectively intervenes in microglial activation and attenuates neuroinflammation by modulating the MAPK/NF-κB signaling pathway.

This study underscores the protective role of TMF in alleviating neuroinflammation, particularly through the inhibition of LPS-activated microglia, and suggests its therapeutic potential in treating the pathology of APP/PS1 mice. However, the precise therapeutic dose of TMF remains uncertain. Similar to the study on luteolin [20], a dose of 40 mg/kg TMF significantly improves AD pathology and cognitive function in APP/PS1 mice, while 10 mg/kg TMF does not yield comparable results. This finding implies that 20 mg/kg may be the minimum effective dose for therapeutic benefit. Further investigation is required in order to fully understand how TMF targets and modulates neuroinflammation and microglial activity. Future research will aim to explore the molecular mechanisms by which TMF addresses neuroinflammation and its broader metabolic impact in AD models.

## 4. Materials and Methods

### 4.1. Reagents and Materials

3′,4′,5,7-tetramethoxyflavone (TMF, purity > 98%) (Figure 1A) was purchased from RENI Pharmaceutical Technology (Chengdu, China). Detailed information on the reagents used can be found in Supplementary Materials Table S1.

### 4.2. Animal Administration

A total of 30 eight-month-old SPF C57BL/6J APPswe/PSEN1de9 (APP/PS1) double transgenic female mice and 10 six-month-old littermate wild-type female mice were purchased from Nanjing Collis Biotechnology Co., Ltd. (Nanjing, China). All mice were housed in SPF classrooms equipped with an IVC automatic air-exchange system, and the temperature was controlled at 24–26 °C and 55–65%, with a 12 h light/dark cycle. All animal experiments were approved by the Ethics Committee of Jiangzhong Pharmaceutical Co., Ltd. (Nanchang, China, Licence No. 20230502) and conducted according to ARRIVE guidelines.

A total of 30 eight-month-old female APP mice (22–25 g) from the same litter were randomly assigned to three groups and given daily oral doses of either 0.2 mL water or TMF at 10 mg/kg and 40 mg/kg. Additionally, 10 wild-type (WT) female mice (22–25 g) received 0.2 mL water daily. Behavioral training began 30 d following the TMF intervention. After all experiments concluded, mice were euthanized, and serum and brain tissues were collected. Three brain samples per group were fixed in 4% PFA, while the other tissues were stored at −80 °C for analysis. Figure 1B shows the experimental procedure.

### 4.3. Behavioural Tests

#### 4.3.1. Morris Water Maze Test

The MWM is a circular tank with a 1 m diameter, equipped with an overhead camera to capture animal behavior. Before starting the experiment, the tank was filled with water at 25 ± 2 °C, and TiO_2_ was added to make the water opaque. The platform was hidden below the water’s surface. In the localization experiment, mice were placed in the water facing the pool wall, allowing them to swim in search of the platform. If the mice failed to find the platform after 60 s, they were guided to it and allowed to stay for 10 s. This process was repeated over five days. In the probe trial, the platform was removed, and each mouse swam freely for 60 s. The data were recorded and analyzed using the EthoVision XT10 (Noldus, Beijing, China).

#### 4.3.2. Open-Field Test

The OFT device includes a white box and a camera to capture animal behavior. The white box measures 40 × 40 cm and is divided into a central area of 20 × 20 cm and a surrounding area. Mice were placed in a consistent starting position facing the wall of the box and observed for 5 min using the Visutrack (Version 3.0) (Xinruan, Shanghai, China). The box was thoroughly cleaned before the next animal was tested.

### 4.4. Immunohistochemistry

Brain sections were dehydrated in graded ethanol. Sections were blocked for 30 min with 5% BSA. After overnight incubation at 4 °C with primary antibodies (diluted 1:200) (Supplementary Materials Table S1), sections were treated with secondary antibodies (diluted 1:1000) and oxidized with 5% DAB. Following DAB staining, images were obtained using a DM3000 microscope (Leica, Hessen, Germany).

### 4.5. Evaluation of Neuroinflammatory Factors

The ELISA assay was used to determine neuroinflammation in the mouse brains. Proteins were extracted with PBS buffer and quantified with the BCA assay (Nanjing Jiancheng Technology, Nanjing, China). Protein levels of TNF-α, IL-1β, and IL-6 (Beyotime, Shanghai, China) were then measured following the manufacturer’s instructions.

### 4.6. Transcriptomics

As in our previous study [51], RNA was extracted and purified. Following purification, reverse transcription to cDNA and second-strand synthesis were completed. Sequencing was conducted on the Illumina NovaSeqTM 6000 platform (Illumina, CA, USA). Data were then filtered and screened, and differential expression analysis was performed with DESeq2 (*p* < 0.05, FC ≥ 2). KEGG and GSEA analysis were used to analyze the pathways associated with DEGs.

### 4.7. Untargeted Metabolomics

The sample was diluted five times with methanol–acetonitrile solution (1:1, *v*/*v*) containing the internal standard for ultrasonic extraction. After incubation, the supernatant was centrifuged (12,000 rpm, 15 min) for analysis, and an aliquot of the supernatant from each mouse sample was made into a quality control sample. Detection was performed using a UPLC chromatography system and an Orbitrap Exploris 120 mass spectrometry system (Thermo Fisher, Waltham, MA, USA), as described in detail in Supplementary Materials Method S1. The resulting data were used for metabolite identification using the R package and BioreeDB (Version 3.0). After data processing, differences in metabolic profiles between groups were assessed by OPLS-DA, and samples were analyzed by clustering. Pathway enrichment analyses were performed using MetaboAnalyst 3.5. A network-based enrichment analysis of pathways, modules, enzymes, reactions, and metabolites was constructed based on the differential metabolites obtained from the previous analysis.

### 4.8. Cell Experiments

#### 4.8.1. Cell Culture

BV2 microglia, obtained from Pricella (Wuhan, China), were cultured in DMEM supplemented with 10% FBS (Gibco, CA, USA) and 1% streptomycin–penicillin (Solarbio, Shanghai, China). Cultures were maintained at 37 °C in a 5% CO_2_ incubator.

#### 4.8.2. Cell Viability and NO Measurement

Cells were seeded at a density of 8 × 10^3^ per well in 96-well plates, pretreated with various concentrations of TMF for 30 min, and then incubated with 1 μg/mL LPS for 24 h at 37 °C. After adding CCK-8 reagent (Beyotime, Shanghai, China) for 2 h, absorbance was measured at 450 nm. For BV2 cells (2 × 10^4^ per well) cultured in 24-well plates, after 24 h of TMF and LPS treatment, the supernatant (3000 rpm, 10 min) was collected via centrifugation and mixed with Griess Reagent (Beyotime, Shanghai, China) as instructed, and absorbance was measured at 540 nm.

#### 4.8.3. mRNA Expression of Inflammatory Factors

Cells were seeded in 6-well plates at a density of 2 × 10^5^ per well and co-incubated with varying concentrations of TMF pretreatment along with LPS. Trizol was used to lyse cells for RNA extraction. Reverse transcription and qPCR were then performed following the instructions provided by the kit manufacturer, and the relative RNA expression of each gene was calculated by the 2^−ΔΔCT^ method. The primer sequences are shown in Supplementary Materials Table S2, where GAPDH is the standardized control.

#### 4.8.4. Immunofluorescence

Cells were seeded at a density of 5 × 10^4^ on cell slides in 12-well plates and treated with TMF pretreatment followed by LPS intervention under the same conditions. After 24 h, the supernatant was aspirated and cells were fixed with 4% PFA at 37 °C for 30 min. Then, cells were permeabilized with 0.2% Triton X-100 at 37 °C for 20 min. Cells were incubated with monoclonal rabbit NF-κB p65 (1:500, Abcam, Cambridge, UK) overnight at 4 °C and then incubated with a secondary antibody for 2 h at 37 °C, protected from light. Nuclei were then stained with DAPI containing fluorescence quenching for 10 min at 37 °C. Fluorescence images were captured with a fluorescence microscope (magnification, ×40).

### 4.9. Western Blot Analysis

Cells were seeded in 6-well plates at a density of 2 × 10^5^, pre-treated with varying concentrations of TMF for 30 min, and then co-incubated with 1 μg/mL LPS for 24 h. Proteins from both treated cells and experimental mouse brain tissues were extracted with RIPA lysis buffer (containing 1% protease and phosphatase inhibitors) and quantified using a BCA assay.

Cell and tissue proteins were separated by SDS-PAGE and transferred onto PVDF membranes, which were blocked with a Western blocking buffer. Membranes were then incubated overnight at 4 °C with primary antibodies. After being washed 3 times with TBST, the blots were incubated with a secondary antibody for 1 h. Visualization was performed with a Super-ECL kit (Yeasen, Shanghai, China) using an automated chemiluminescence imaging system, and bands were quantified using Image J (Version 6.0) (NIH, Bethesda, MD, USA).

### 4.10. Statistical Analysis

All experimental results were presented as the mean ± standard error of the mean (SEM). One-way ANOVA with Tukey’s post hoc test was used for group comparisons, while two-way ANOVA was applied to analyze WMW escape latency. Student’s *t*-test was used for comparisons between omics groups. Statistical analyses were performed using GraphPad Prism 8.0 software (San Diego, CA, USA), with statistical significance set at *p* < 0.05.

## 5. Conclusions

In conclusion, this study demonstrates the potential of TMF as a therapeutic agent for AD by effectively mitigating neuroinflammation and addressing core pathological features such as Aβ plaques, NFTs formation, and cognitive deficits. TMF demonstrated significant neuroprotective effects in the APP/PS1 mice, as evidenced by its ability to counter neuroinflammation, reduce microglial and astrocyte activation, and inhibit the MAPK and NF-κB pathways. Additionally, the multi-omics analysis provided insights into the molecular mechanisms underlying TMF’s effects, including its role in modulating arginine and proline metabolism. The consistency of these findings in in vivo and in vitro models highlights that TMF has a significant neuroprotective effect.

## Data Availability

The data will be made available upon request.

## Supplementary Materials

The following supporting information can be downloaded at: www.mdpi.com/article/10.3390/md23020074/s1, Table S1: Reagents and drugs; Table S2: qPCR primer sequence; Table S3: The top 10 pathways in the Metabolomics Interactive Network; Method 1: The elution conditions and mass spectrometry conditions in LC-MS/MS analysis in metabolomics.
