# Supplementary material for: TMF Attenuates Cognitive Impairment and Neuroinflammation by Inhibiting the MAPK/NF-κB Pathway in Alzheimer’s Disease: A Multi-Omics Analysis
Source: Mar Drugs. 2025 Feb 7;23(2):74. doi: 10.3390/md23020074 (PMC11857128; doi:10.3390/md23020074)
Supplement: Supplementary file 1 [file marinedrugs-23-00074-s001.zip › marinedrugs-3444109-supplementary.pdf]

Supplementary Material

Supplementary Table S1. Reagents and drugs

Name	Dilution Ratio	Catalog Number	Producer	Western Blotting Conditions
Primary Antibody Dilution Buffer	-	P0256	Beyotime (Shanghai, China)	-
Secondary Antibody Dilution Buffer	-	P0258	Beyotime (Shanghai, China)	-
Western Blocking Buffer	-	P0252	Beyotime (Shanghai, China)	-
Beta Amyloid	1:1000	25524-1-AP	Proteintech (Wuhan, China)	Block 1 h
p-tau (S396)	1:2000	AB32057	Abcam (USA)	Block 2 h
Tau	1:1000	66499-1-Ig	Proteintech (Wuhan, China)	Block 0.5h
Iba1	1:1000	10904-1-AP	Proteintech (Wuhan, China)	Block 2 h
GFAP	1:1000	Bs-0199R	Bioss (Beijing, China)	Block 2 h
p38 MAPK	1:2000	8690	CST (USA)	Block 0.5h
Phospho-p38 MAPK	1:2000	4511	CST (USA)	Block 1 h
JNK	1:2000	9252	CST (USA)	Block 0.5h
Phospho-JNK	1:2000	4668	CST (USA)	Block 1 h
ERK	1:2000	ab184699	Abcam (USA)	Block 0.5 h
Phospho-ERK	1:2000	ab201015	Abcam (USA)	Block 1 h
NF-κB p65	1:2000	ab32536	Abcam (USA)	Block 0.5 h
Phospho-NF-κB p65	1:2000	ab76302	Abcam (USA)	Block 1 h
Beta Actin	1:5000	20536-1-AP	Proteintech (Wuhan, China)	Block 0.5 h
HRP-conjugated Goat Anti-Rabbit IgG(H+L)	1:10000	SA00001-2	Proteintech (Wuhan, China)	-
Alexa Fluor 594-conjugated goat anti-rabbit	1:5000	ab150080	Abcam (USA)	
Mouse TNF-α ELISA Kit	-	PT512	Beyotime (Shanghai, China)	-
Mouse IL-1β ELISA Kit	-	PI301	Beyotime (Shanghai, China)	-
Mouse IL-6 ELISA Kit	-	PI326	Beyotime (Shanghai, China)	-
PBS	-	P1020	Solarbio (Beijing, China)	-
Trypsin-EDTA solution	-	T1300	Solarbio (Beijing, China)	-
Cell Counting Kit-8	-	C0039	Beyotime (Shanghai, China)	-
Nitric Oxide Assay Kit	-	S0021M	Beyotime (Shanghai, China)	-
Fetal Bovine Serum	-	10091148	Gibco (USA)	-
Dexamethasone	-	D4902	Sigma-Aldrich (USA)	-

Supplementary Table S2. qPCR primer sequence

Gene		5' to 3'
GAPDH	Forward	5'- AGAAGGTGGTGAAGCAGGCATCT -3'
	Reverse	5'- CGGCATCGAAGGTGGAAGAGTG -3'
iNOS	Forward	5'- TTGCCACGGACGAGACGGATA -3'
	Reverse	5'- AACTCTTCAAGCACCTCCAGGAAC -3'
COX-2	Forward	5'- GGTGCCTGGTCTGATGATGTATGC -3'
	Reverse	5'- GAAGTGGTAACCGCTCAGGTGTTG -3'
IL-6	Forward	5'- AAGAGACTTCCATCCAGTTGCCTTC -3'
	Reverse	5'- TGTGTAATTAAGCCTCCGACTTGTGA -3'
TNF-α	Forward	5'- GGAACTGGCAGAAGAGGCACTC -3'
	Reverse	5'- GCAGGAATGAGAAGAGGCTGAGAC -3'

Supplementary Table S3. The top 10 pathways in the Metabolomics Interactive Network

KEGG ID	Entry type	KEGG name
mmu00220	pathway	Arginine biosynthesis
mmu00360	pathway	Phenylalanine metabolism
mmu03320	pathway	PPAR signaling pathway
mmu04010	pathway	MAPK signaling pathway
mmu04014	pathway	Ras signaling pathway
mmu04022	pathway	cGMP-PKG signaling pathway
mmu04024	pathway	cAMP signaling pathway
mmu04340	pathway	Hedgehog signaling pathway
mmu04670	pathway	Leukocyte transendothelial migration
mmu04710	pathway	Circadian rhythm

Supplementary Method S1. The elution conditions and mass spectrometry conditions in LC-MS/MS analysis in untargeted metabolomics

For polar metabolites, LC-MS/MS analyses were performed using an UHPLC system (Vanquish, Thermo Fisher Scientific, USA) with a Waters ACQUITY UPLC BEH Amide (2.1 mm × 50 mm, 1.7 μm) coupled to Orbitrap Exploris 120 mass spectrometer (Orbitrap MS, Thermo Fisher Scientific, USA). The mobilephase consisted of 25 mM ammonium acetate and 25 ammonia hydroxide in water (pH = 9.75) (A) and acetonitrile (B). The auto-sampler temperature was 4 ℃, and the injection volume was 2 μL. The Orbitrap Exploris 120 mass spectrometer was used for its ability to acquire MS/MS spectra on information-dependent acquisition (IDA) mode in the control of the acquisition software (Xcalibur, Thermo Fisher Scientific, USA). In this mode, the acquisition software continuously evaluates the full scan MS spectrum. The ESI source conditions were set as following: sheath gas flow rate as 50 Arb, Aux gas flow rate as 15 Arb, capillary temperature 320 ℃, full MS resolution as 60000, MS/MS resolution as 15000, collision energy: SNCE 20/30/40, spray voltage as 3.8 kV (positive) or -3.4 kV (negative), respectively.
